# The Impact of Storage Conditions and Bottle Orientation on the Evolution of Phenolic and Volatile Compounds of Vintage Port Wine

**DOI:** 10.3390/foods11182770

**Published:** 2022-09-08

**Authors:** Joana Azevedo, Joana Pinto, Natércia Teixeira, Joana Oliveira, Miguel Cabral, Paula Guedes de Pinho, Paulo Lopes, Nuno Mateus, Victor de Freitas

**Affiliations:** 1 LAQV—REQUIMTE/Laboratório Associado para a Química Verde, Faculdade de Ciências, Universidade do Porto, Rua do Campo Alegre, 687, 4169-007 Porto, Portugal; 2 Associate Laboratory i4HB—Institute for Health and Bioeconomy, Faculdade de Farmácia, Universidade do Porto, Rua Jorge Viterbo Ferreira, 228, 4050-313 Porto, Portugal; 3 UCIBIO—REQUIMTE, Laboratório de Toxicologia, Departamento de Ciências Biológicas, Faculdade de Farmácia, Universidade do Porto, Rua Jorge Viterbo Ferreira, 228, 4050-313 Porto, Portugal; 4 Amorim Cork S.A., Rua dos Corticeiros, 830, 4536-904 Santa Maria de Lamas, Portugal

**Keywords:** Vintage Port wine, phenolic compounds, volatile compounds, cellar conditions, bottle orientation, cork

## Abstract

This work evaluates the influence of the cellar conditions and bottle orientation, on the phenolic and volatile composition of a Vintage Port wine, sealed with natural cork stoppers, for 44 months post-bottling. The storage was performed in two different cellars, namely a cellar A with controlled temperature and humidity, and a cellar B, representing a traditional cellar, with uncontrolled temperature and humidity. The impact of bottle orientation was studied in cellar A, where the bottles were stored in horizontal and vertical positions. The phenolic and volatile composition of the bottled Vintage Port wine were analyzed after 6, 15 and 44 months. The results unveiled that the cellar conditions and bottle orientation had an impact in Port wine composition which was higher at 44 months post-bottling. The samples stored in the traditional cellar unveiled significantly higher yellow tones, lower tannin specific activity, and higher levels of furfural and 5-methylfurfural. Furthermore, the samples stored in the horizontal position revealed significant higher levels of total proanthocyanidins and higher tannin specific activity than the samples stored in the vertical position. Interestingly, for the first time to our knowledge, an ellagitannin-derived compound (Corklin) was detected in Vintage Port wines stored in the horizontal position, which results from the reaction of cork constituents with phenolic compounds present in wines.

## 1. Introduction

Port wine is a fortified wine produced in the Douro region of Portugal [1]. There are several special categories of Port wine according to the winemaking processes (e.g., Vintage, Ruby, Tawny, among others). The term Vintage is only attributed to Port wine with superior quality and structure that is made from grapes of a single vintage. This type of Port wine is matured in wood for 2–3 years followed by bottle ageing for 10 to 50 years or more before consumption. During bottle ageing, the polyphenolic and volatile profiles of Port wine undergo important transformations having an impact in the commercial value of aged Vintage Port wine. Thus, the storage conditions, namely temperature and oxygen (O_2_) exposure, are critical for color and aroma characteristics of these wines as they directly influence oxidative transformations during wine ageing [2,3]. Indeed, some studies have investigated the influence of dissolved O_2_ and free SO_2_ levels, pH, and time/temperature on the volatile profile of Port wine. Among these effects, temperature and pH had the largest impact leading to the formation of several volatiles such as 5-methylfurfural, furfural, sotolon, acetaldehyde, heterocyclic acetals [4,5], glyoxal and methylglyoxal [6]. Moreover, the aldehydes present in Port wine can be involved in the condensation processes of anthocyanins with proanthocyanidins having an impact in the color stability and evolution [7,8]. In line with this, the decrease on polyphenols during wine ageing has been reported in the literature and can be due to the chemical transformation, polymerization, complexation, and degradation phenomena’s [9,10,11,12,13,14,15,16]. Furthermore, it has been reported that when cork is in contact with an aqueous solution of ethanol, such as the case of Port wine, phenolic and volatile compounds can be extracted from cork [17,18] and can participate in the same interactions.

Considering the importance of storage conditions for the quality of bottle-aged Vintage Port wine, the aim of this work was to evaluate the influence of cellar conditions (controlled and uncontrolled temperature and humidity) and bottle orientation (horizontal and vertical) on the phenolic and volatile composition of a Vintage Port wine sealed with a natural cork stopper, over a period of 44 months. This work also had the purpose to detect and identify compounds that could arise from the interaction of cork with wine in the horizontal position. A better understanding of the impact of cellar conditions and bottle orientation on the physical-chemical properties of Vintage Port is of extreme importance for the Port wine industry to select the most appropriate storage conditions to produce high quality wines.

## 2. Materials and Methods

### 2.1. Reagents

The L-(+)-tartaric acid (99%), ethyl acetate (99.9%), methanol (99.8%), acetonitrile (99.8%), acetic acid (99.7%), Folin–Ciocalteu reagent, and sodium bisulfite were obtained from Sigma-Aldrich, Madrid, Spain. Ethanol was purchased from AGA^®^ (96%), Prior Velho, Portugal and HCl 37% from Fluka^®^, College Park, MD, USA. All standard compounds used in the quantification of volatiles presented high purity (≥95%) as described elsewhere [19] and were purchased from Sigma-Aldrich, Inc. (Steinheim, Germany).

### 2.2. Port Wine Samples

The Port wine used in this study was a Vintage Port Wine 2014 produced with a blend from several varieties from Douro region (*Touriga Franca*, *Touriga Nacional*, *Tinta Barroca*, *Tinta Roriz* and *Tinto Cão*) using the traditional method for Vintage Port wine. The wine presented the following chemical characteristics: alcohol % vol. (20 °C): 19.73; density (g/L): 1022.0; baume: 3.37; color 420 nm (1 mm PL): 0.888; color 520 nm (1 mm PL): 1.521; total SO_2_: 90.0 mg/L. Forty-six bottles (Vintage model 1790 of a single batch of manufacture) were filled with the Port wine obtained from the same tank and sealed with natural cork stoppers of grade A 49 × 24 mm from the same origin (provided by Amorim^®^ Cork S.A.). The study was designed to maintain the homogeneity of wine samples, with the physical-chemical features being only affected by the two factors under study, namely the cellar conditions and bottle orientation. The characteristics of the cellars used in this study are presented in Table 1. Cellar A had air conditioning and consequently minor variations of temperature and humidity, while Cellar B represents a traditional cellar without any humidity and temperature control, allowing the wine to suffer temperature fluctuations according to the season of year. All bottles were protected from light in both cellars. In cellar A, half of the total bottles were stored in vertical position and the other half in the horizontal position, whereas in Cellar B all bottles were stored in horizontal position. At each analysis time (0, 6, 15, 44 months), five bottles from each condition were analyzed: Cellar A vertical, Cellar A horizontal, and Cellar B horizontal.

### 2.3. Color Index (CIELAB, Color Index and Hue)

The color characteristics were evaluated by the chromatic intensity (CI), color hue and CIELAB color coordinates (L^∗^, C^∗^, H^∗^), where L^*^ is the lightness, C^*^ the chromaticity and H° the hue, measured by direct absorption and determined by Color Win-MSCV^®^ Coordinates software. For that, the absorbance (Abs) at 450, 520, 570 and 630 nm were determined for all samples using a 2 mm optical path cuvette [20]. For the color index determination, the Abs at 420, 520 and 620 nm of all wine samples were determined using a 1 mm cell. The hue was determined by the ratio between the Abs at 420 and 520 nm. All determinations were performed on a UV–vis spectrophotometer Thermo^®^ Scientific Evolution Array UV–vis spectrophotometer (ThermoFisher Scientific, Waltham, MA, USA).

### 2.4. Total Phenolics Index

The relative amount of total phenolic compounds between samples was estimated by recording the Abs at 280 nm of diluted wine samples (10 times with wine model solution, 12% ethanol, 5 g/L tartaric acid, pH 3.5) using a quartz cell of 1 mm [21,22].

### 2.5. Total Proanthocyanidins

The total proanthocyanidins (condensed tannins) were determined based on the Bate-Smith reaction [23]. This method consists on the acidic decomposition of condensed tannins in the presence of heat (100 °C) in strongly acidic conditions yielding to anthocyanidins that can be determined at 520 nm [24]. As Port wines contain a high concentration of sugar and to avoid caramelization reactions that will have an impact on the Abs at 520 nm, samples were pre-treated to remove sugars. This operation consisted in the application of 1 mL of Port wine in a C18 gel cartridge^®^ which was washed with distilled water, and then the wine fraction without sugar was recovered with 4 mL of methanol. Methanol was evaporated and the sample resuspended in 1 mL of distilled water. Then, each wine sample was diluted 50 times with a wine model solution (tartaric acid 5 g/L, 12% ethanol, pH 3.5) and 4.0 mL of these diluted wine plus 2.0 mL of water and 6.0 mL of HCl (37%) were placed into a hydrolysis tube and heated to 100 °C for 30 min. The tubes were cooled in water, protected from light for 10 min and then, 1.0 mL of ethanol was added and mixed. The procedure was performed in triplicate for each sample and reference was obtained using the same procedure but in the absence of heat. The Abs at 520 nm of all samples was recorded using a 1 cm glass cell in a Thermo^®^ Evolution Array spectrophotometer (ThermoFisher, Waltham, MA, USA). The change in Abs is calculated by the difference between the Abs of the sample and the Abs of the reference tube, according to the equation:

ΔAbs = Abs (520 nm) _sample_ − Abs (520 nm) _reference_

The concentration of condensed tannins in wines was determined through a calibration curve (R^2^ = 0.9911) obtained using different concentrations of procyanidins ranging from 0.264–5.92 mg/mL. Procyanidin standards were comprised by a fraction of dimeric and trimeric procyanidins purified from grape seeds [23,25].

### 2.6. Total Free Anthocyanins

The concentration of free anthocyanins was determined using the procedure described in the literature [8], by adaptation of the Sommers & Evans method [26]. Briefly, 40.0 mL of HCl 2%, 2.0 mL of ethanol and 2.0 mL of wine were added to a glass flask. Then, 10.0 mL of the mixture was added to four glass tubes and then 4.0 mL of sodium bisulfite 20% (*w*/*v*) was added to three of them. To the fourth tube (reference), 4.0 mL of water was added. The samples were vortexed and left in the dark during 20 min. The Abs at 520 nm was recorded for all the samples using a 1 cm glass cell in a Thermo^®^ UV-VIS Evolution Array Spectrophotometer (Thermo Fisher Scientific, Waltham, MA, USA). The concentration of the free anthocyanins in wine corresponds to the difference between the Abs of the samples and the reference using the calibration curve prepared with malvidin-3-*O*-glucoside, according to the equations:

ΔAbs = Abs (520 nm) _reference_ − Abs (520 nm) _sample_

[Anthocyanins _Wine_]_mg/L_ = (Δabs − 0.00024)/0.0015

### 2.7. Dialysis Index

This assay was performed according to the procedure described in the literature [27,28]. Briefly, 5.0 mL of wine was introduced into a dialysis tubing (cellulose; 6 mm i.d. nominal molecular weight cut-off 12.000–16.000; average porous radius of 25 Å) and then submerged in a glass bottle with 50.0 mL of 12% aqueous ethanol solution (5 g/L tartaric acid, pH 3.5). In a second bottle, 5.00 mL of wine was diluted directly with the same hydroalcoholic solution up to 50.0 mL (reference). Both glass bottles were closed and stored at room temperature during 24 h. Then, the Abs at 280 nm was determined in a 1 mm quartz cell. The difference between the Abs at 280 nm of the reference and the sample correspond to the fraction of more polymerized phenolic compounds that were retained inside the dialysis membrane.

### 2.8. Tannin Specific Activity

The Tannin Specific Activity (TSA) determines the capacity of phenolic compounds to precipitate a protein (bovine serum albumin, BSA) and it is measured by turbidimetry as described elsewhere [25,29]. Red wines were diluted to 1:50 with a filtered (0.45 μm) wine model solution (12% ethanol, 5.0 g/L tartaric acid, pH 3.50). Then, 4.0 mL of this solution were transferred to a turbidimetry tube and the turbidimetry values were recorded in Turbidimeter HACH 2100 N (HACH, Lisbon, Portugal) adapted for cells of 100 × 12 mm. Then, 150 μL of BSA (0.8 mg/mL) was added to each tube and vortexed. The tubes were kept in the dark during 30 min, and then, the maximum turbidity was determined. The TSA was expressed in turbidity units NTU/mL of wine and determined by the following expression, where 0.08 corresponds to the dilution factor of wine: Turbidity (NTU/_mL of wine_) = (Turbidity _after BSA_ − Turbidity t_0_)/0.08.

### 2.9. Volatile Composition

The concentration of 36 volatile compounds in Porto Wine was determined by headspace solid-phase microextraction (HS-SPME) coupled to gas chromatography-mass spectrometry (GC-MS) using a method adapted from Barros et al. [30]. The volatile compounds included several alcohols, aldehydes, ethyl esters, ketones, and isoprenoids. Briefly, 250 µL of each wine were placed in a 20 mL glass vial and the HS-SPME procedure was carried out using a Combi-PAL autosampler (Varian Pal Autosampler, Zwingen, Switzerland). The GC-MS analysis was performed in a 436-GC model coupled to a SCION single quadrupole (SQ) mass spectrometer (Bruker Daltonics, Bremen, Germany) and a Bruker Daltonics MS workstation (version 8.2.1, Bruker Daltonics, Bremen, Germany). The HS-SPME and GC-MS conditions considered for analysis of Porto Wine are described in detail in our previous studies [19,31]. The calibration curves determined for quantification of volatile compounds were achieved by analysis, under the same analytical conditions, of standard compounds dissolved in a wine model solution (12% ethanol, 5 g/L of tartaric acid, pH 3.2) in a range of known concentrations.

### 2.10. Identification of Cork Phenolic Compounds Extracted to Port Wines

#### 2.10.1. Wine Treatment

The first step was the elimination of ethanol from the Port wine, under vacuum using rotary evaporator. After that, 100 mL of Port Wine were applied into a C18 gel (using a G3 funnel with Buchner and vacuum system) and the aqueous phase was collected (1 L). This fraction was then evaporated under vacuum to about 200 mL of the final volume. The aqueous fraction was extracted with ethyl acetate three times (80 mL) and then all organic fractions were combined and the organic solvent evaporated. The solid phase was resuspended in 5 mL methanol/water (1:1) and then analyzed by LC-ESI-MS.

#### 2.10.2. Detection of Corklin by Liquid Chromatography-DAD/Electron Spray Ionization (ESI)-Mass Spectrometry (MS) Analysis

A Finnigan Surveyor series liquid chromatograph (Thermo Fisher Scientific, Waltham, MA, USA), equipped with a Thermo Finnigan (Hypersil Gold^®^) reversed-phase column (150 mm × 4.6 mm, 5 μm, C18) thermostated at 25 °C was used. The samples were analyzed using the same solvents, gradient, injection volume, and flow rate referred above for the HPLC analysis. Double-online detection was done by a photodiode spectrophotometer and mass spectrometer (Thermo Fisher Scientific, Waltham, MA, USA). The mass detector was a Finnigan LCQ DECA XP MAX (Finnigan Corp., San Jose, CA, USA) quadrupole ion trap equipped with atmospheric pressure ionization (API) source, using electrospray ionization (ESI) interface. The vaporizer and the capillary voltages were 5 kV and 4 V, respectively. The capillary temperature was set at 325 °C. Nitrogen was used as both sheath and auxiliary gas at flow rates of 80 and 30, respectively (in arbitrary units). Spectra were recorded in the negative ion mode between *m*/*z* 120 and 2000. The mass spectrometer was programmed to perform a series of three scans: a full mass, a zoom scan of the most intense ion in the first scan, and a MS-MS of the most intense ion using relative collision energies of 30 and 60.

### 2.11. Statistical Analysis

All determinations were performed in triplicated, except for volatile composition. Values are expressed as the arithmetic means ± standard deviation. Statistical significance of the difference between the wines was evaluated by ANOVA followed by Tukey test for multiple testing correction, using GraphPad Prism (version 9.3.0, GraphPad Software, San Diego, CA, USA).

## 3. Results and Discussion

### 3.1. Effect of the Cellar Conditions

The effect of the cellar conditions (temperature and humidity) on the phenolic and volatile composition of Vintage Port wine was studied over a period of 44 months. As shown in Table 1, the cellar A presented a lower thermic amplitude and a higher medium humidity due to the presence of air conditioner. On the other hand, the cellar B was under a higher thermic amplitude, with a much higher maximum temperature when compared with cellar A, namely 33 °C versus 22 °C, respectively (Table 1). Table 2 presents the statistically significant alterations occurring in color, phenolic and volatile composition of Porto wine induced by cellar conditions. These results showed that the main differences in color and phenolic composition between both cellars were in the wines b* CIELAB coordinate, hue, and tannin specific activity. Wines stored at cellar B displayed higher hues and b* values after 44 months of storage, indicating more yellow tones than the wines stored at cellar A. This result is in agreement with a recent study showing that “Verdejo” white wines stored under commercial conditions (diffuse white LEDs and temperature of 24 ± 2 °C) revealed higher levels of brown-yellowish hues when compared with wines stored in the dark at 12 °C [32]. The comparison of the phenolic profile of red wines under different storage conditions (controlled cellar and typical domestic conditions) [33] also revealed that the controlled cellar (15–17 °C, ~70% humidity) preserved better the chemical and organoleptic characteristics of the wines even after 2 years of storage, while the wines stored in typical domestic conditions (20–27 °C, uncontrolled humidity) aged approximately four times faster.

Furthermore, the tannin specific activity data showed that the wines stored at cellar A presented a higher ability to interact with proteins which may indicate a higher astringency.

**Figure 1:**
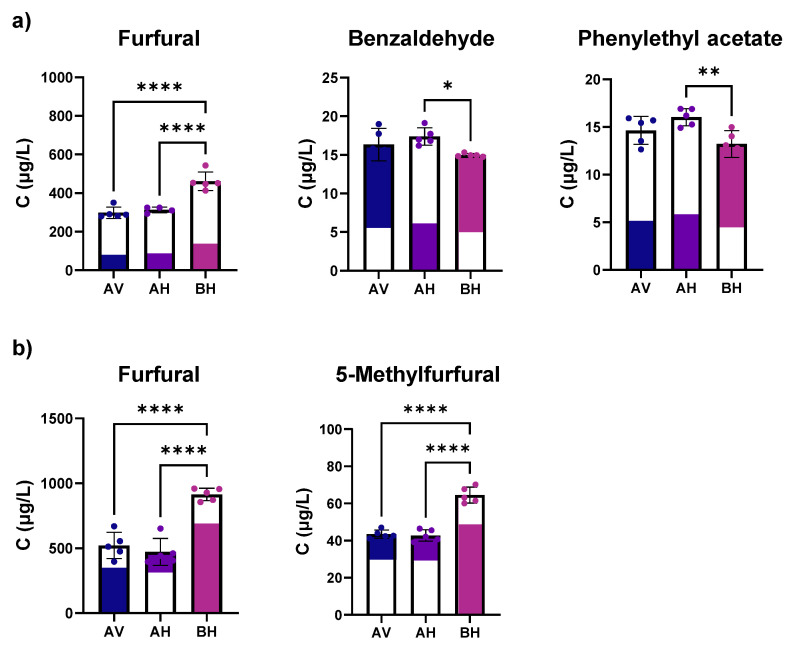
Boxplots illustrating the levels of volatile compounds present in Vintage Port wine that changed with the cellar conditions at (**a**) 15 months and (**b**) 44 months. AV—cellar A vertical position, AH—cellar A horizontal position, BH—cellar B horizontal position. *—*p* ≤ 0.05, **—*p* ≤ 0.01, ****—*p* ≤ 0.0001.

Regarding the impact of cellar conditions on the volatile composition of Vintage Port wine, among the 36 volatile compounds determined (Table S2), five showed significant alterations between the two cellars at 15- or 44-months post-bottling (Table 2, Figure 1). At 15 months post-bottling, significant higher levels of furfural and significant lower levels of benzaldehyde and phenylethyl acetate were found in wines stored in cellar B compared with A (Figure 1a). The results found at 44 months confirmed the significant increase of furfural in cellar B (Table 2, Figure 1b), in tandem with another furan compound only determined at this time point (5-methylfurfural).

The remaining 34 volatile compounds showed relatively constant levels between both cellars across the bottle ageing period (Table S2). The increase observed in the levels of furfural and 5-methylfufural in cellar B may be due to prolonged storage at higher and uncontrolled temperature which can lead to the occurrence of Maillard reaction, caramelization, and an increased susceptibility to oxidation [34,35]. Furfural and 5-methylfurfural were present in concentrations above the human olfactory perception threshold (Table 2) and typically contribute to toasty and caramel aroma in wines [36,37,38]. The increase in the levels of both furan compounds with temperature was previously observed by Silva Ferreira et al. [5], but considering Port wine under forced ageing conditions (60 °C temperature and different oxygen saturations). The decrease observed in the levels of benzaldehyde may be related with its use in the formation of condensed pigments through the reaction between anthocyanins, flavanols and benzaldehyde [39]. Finally, the decrease of phenylethyl acetate levels with the increase of storage temperature was already reported for Cabernet Sauvignon Wine [40] and may be attributed to hydrolysis.

### 3.2. Effect of the Bottle Storage Position

To study the effect of the bottle storage position on the physical-chemical parameters of Vintage Port wine, the bottles were stored in the cellar A in vertical and horizontal positions. The statistically significant alterations found in Port wine stored with different bottle positions are also presented in Table 2. Significant alterations were noted in the total proanthocyanidins at 15 months, tannin specific activity at 6 and 44 months, and benzaldehyde at 44 months. Regarding the color parameters determined by the CIELAB system, the results obtained for the wines showed no significant differences for L*, a*, b*, C*, Hº, Hue and CI parameters (Table S1). For that reason, we can assume that the bottle position didn’t have an impact on the final color of wines, at least for 44 months.

At 15 months, the levels of total proanthocyanidins were significant higher in the Porto wine stored in the horizontal position when compared to the wines stored in the vertical position (Table 2). After 15 months and up to 44 months, it was observed an important decrease in proanthocyanins levels, for all wines regardless of the position. The tannin specific activity (TSA) of Porto wine was affected by bottle position at 6 and 44 months showing a contradictory tendency since the TSA was significantly higher in the wine stored in the vertical position at 6 months and significantly higher in the wine stored in the horizontal position at 44 months. These results combined with those of total proanthocyanidins suggest that wines stored in horizontal position may have more complex and astringent structures, and therefore, higher capacity to interact with salivary proteins [25]. Moreover, a study investigating the effect of different sealing and storage conditions revealed that white and red wines were better preserved when the bottles were keeping horizontally, according to chemical parameters and sensory analysis [41].

Regarding the volatile composition of Vintage Port, the bottle position only showed an influence in the levels of benzaldehyde which were found significantly lower in wines stored in the horizontal position at 44 months post-bottling (Table 2). No significant differences were observed in the levels of the remained 35 volatile compounds between different bottle positions (Table S2). As mentioned above, benzaldehyde is a very reactive molecule that can participate in the formation of condensed pigments by reaction with polyphenols [39]. In fact, the horizontal position enables a direct contact of Vintage Port wine with the natural cork stopper which can promote the occurrence of reactions between aldehydes and phenolic compounds present in cork.

**Figure 2:**
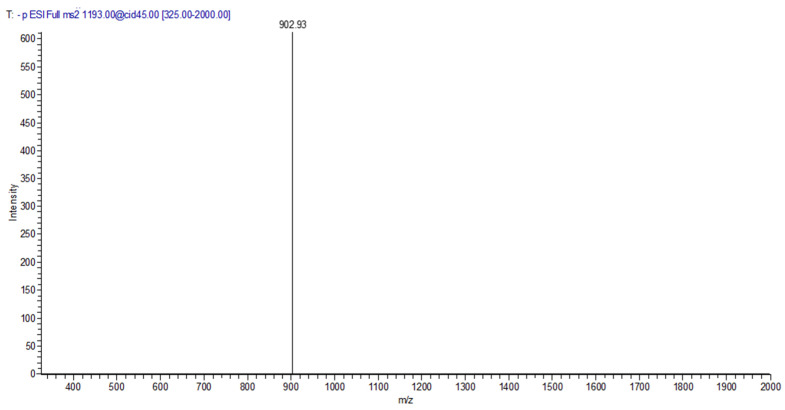
MS^2^ spectrum of Corklin (*m*/*z* 1193) obtained by LC MS analysis of Vintage Port wine stored in the horizontal position, with the main fragment at *m*/*z* 903 (M—290, loss of a catechin unit).

Since previous studies of our group proved that cork compounds can react with main wine constituents, namely (+)-catechin and malvidin-3-*O*-glucoside giving origin to new compound classes, such as Corklins [42], the presence of these compounds was investigated in all bottled wine samples studied at 44 months of storage by LC-MS analysis. Indeed, it was possible to detect a compound with an ion mass at *m*/*z* 1193 and a fragment at *m*/*z* 903 (M − 290, loss of a catechin unit) (Figure 2) in trace amounts in wines stored in the horizontal position (Table S3). The fragmentation pattern and the retention time of the detected peak agrees with the data previously reported for Corklin [42]. This compound results from the interaction between vescalagin (extracted from cork) ethanol derivative and (+)-catechin present in the wine. These findings suggest that the direct contact between Vintage Port wine and cork during the horizontal storage promotes the formation of Corklins.

## 4. Conclusions

This study unveiled that a Vintage Port wine 2014 stored in a traditional cellar (cellar B) under larger thermic amplitudes over the year had more yellow tones (higher hues and b* values), lower astringency (lower tannin specific activity), lower levels of phenylethyl acetate, and higher levels of oxidation markers (furfural and 5-methylfurfural) contributing for toasty and caramel aromas, when compared with the same Port wine stored in a cellar with controlled temperature (cellar A). Importantly, the impact of larger thermic amplitudes on the tannin specific activity and the phenylethyl acetate levels of Port wine was observed in this work for the first time. Moreover, the Vintage Port stored in the horizontal position showed for the first time, to our knowledge, higher levels of total proanthocyanidins, more complex and astringent structures (higher tannin specific activity), the presence of one compound derived from the reaction of wine and cork components (Corklin) and lower levels of benzaldehyde, when compared with the same wine stored in the vertical position. Overall, these findings were more evident at 44 months post-bottling. The results obtained in this study are of extreme importance for the Port Wine Industry providing new insights for the selection of cellar conditions and bottle orientation according to the desired characteristics for a Vintage Port wine.

## Figures and Tables

**Table 1:** Average, maximum, minimum and amplitude of temperature and humidity recorded in the Cellars A and B during the period under study (44 months).

Local	Temperature (°C)/Humidity (%)			
	Average	Maximum	Minimum	Amplitude ^a^
Cellar A	18/60	22/81	14/40	1/5
Cellar B	23/58	33/85	13/34	3/8

^a^ The amplitude indicates the difference between the average highest temperature/humidity and the average lowest temperature/humidity in the studied time period.

**Table 2:** Color, phenolic parameters and volatile composition of Vintage Port wine stored under different cellar conditions and bottle orientation for 44 months in bottle (*n* = 5).

Parameters/ Volatiles	Months	Cellar A		Cellar B	Statistical Differences	Descriptor (Olfactory Threshold)
		Vertical	Horizontal	Horizontal		
b*	T = 0	28	28	28	-	-
	6	16.0 (6.6)	19.7 (3.1)	21.1 (3.7)	**n.s.**	
	15	26.4 (2.6)	26.6 (0.9)	26.4 (1.3)	**n.s.**	
	44	19.1 (0.7)	18.2 (2.1) **a**	25.1 (13.7) **a**	*	
**Hue** **(Abs 420 nm/Abs 520 nm)**	T = 0	0.58	0.58	0.58	-	-
	6	0.74 (0.08)	0.79 (0.05)	0.70 (0.04)	**n.s.**	
	15	0.64 (0.06)	0.61 (0.02)	0.63 (0.03)	**n.s.**	
	44	0.72 (0.06)	0.65 (0.14) **a**	0.78 (0.06) **a**	**	
**Total Proanthocyanidins (g/L)**	T = 0	3.2	3.2	3.2	-	-
	6	2.8 (0.3)	3.1 (0.3)	3.3 (0.2)	**n.s.**	
	15	2.9 (0.3) **a**	3.5 (0.3) **a**	3.3 (0.2)	*	
	44	1.3 (0.5)	1.3 (0.1)	0.8 (0.5)	**n.s.**	
**Tannin Specific Activity (NTU/mL)**	T = 0	404	404	404		-
	6	392.1 (10.6) **a**	360.8 (13.0) **a**	375.1 (12.5)	**	
	15	361.1 (9.4)	342.1 (12.0)	347.8 (19.3)	**n.s.**	
	44	411.1 (17.2) **a, b**	471.5 (29.2) **a**	436.3 (15.3) **b**	***	
**Furfural (µg/L)**	T = 0	137	137	137	-	Toasty, caramel (14 µg/L)
	6	207.6 (73.7)	227.0 (97.8)	308.0 (118.2)	**n.s.**	
	15	298.2 (26.4) **a**	359.0 (92.2) **b**	461.4 (43.3) **a, b**	****	
	44	521.4 (90.0) **a**	471.3 (93.0) **b**	914.1 (42.7) **a, b**	****	
**5-Methylfurfural (µg/L)**	T = 0	NQ	NQ	NQ	-	Spicy, toasty (16 µg/L)
	6	NQ	NQ	NQ	-	
	15	NQ	NQ	NQ	-	
	44	43.5 (2.0) **a**	42.8 (2.8) **b**	64.5 (3.9) **a, b**	****	
**Benzaldehyde (µg/L)**	T = 0	45	45	45	-	Bitter almonds (2 mg/L)
	6	34.4 (10.4)	35.7 (8.3)	33.9 (8.8)	**n.s.**	
	15	16.3 (1.9)	17.4 (1.0) **a**	15.0 (0.2) **a**	*	
	44	32.7 (4.7) **a, b**	25.4 (2.9) **a, b**	28.9 (3.0) **a**	*	
**Phenylethyl acetate (µg/L)**	T = 0	NQ	NQ	NQ	-	Floral, sweet (250 µg/L)
	6	NQ	NQ	NQ	-	
	15	14.6 (1.3)	16.0 (0.8) **a**	13.2 (1.3) **a**	**	
	44	32.2 (1.1)	32.8 (1.7)	30.8 (1.2)	**n.s.**	

Results expressed as an average (standard deviation) of three readings for each bottle. Identical letters indicate statistically significant differences among samples stored under different cellar conditions and bottle orientation within the same post-bottling period. n.s. *p* > 0.05, * *p* ≤ 0.05, ** *p* ≤ 0.01, *** *p* ≤ 0.001, **** *p* ≤ 0.0001, NQ not quantified.

## Data Availability

The data presented in this study are available in supplementary material.

## Supplementary Materials

The following supporting information can be downloaded at: https://www.mdpi.com/article/10.3390/foods11182770/s1, Table S1. Color and phenolic parameters of Vintage Port wine stored under different cellar conditions and bottle orientation at 6-, 15- and 44-months post-bottling (*n* = 5 per group at each time point), Table S2. List of the 36 volatile compounds quantified in Vintage Porto Wine under different cellar conditions and bottle orientation at 6-, 15- and 44-months post-bottling (*n* = 5 per group at each time point), Table S3: List of Vintage Port wine bottles where Corklin was detected at 44 months post-bottling.
