# Supplementary material for: The Impact of Storage Conditions and Bottle Orientation on the Evolution of Phenolic and Volatile Compounds of Vintage Port Wine
Source: Foods. 2022 Sep 8;11(18):2770. doi: 10.3390/foods11182770 (PMC9498223; doi:10.3390/foods11182770)
Supplement: Supplementary file 1 [file foods-11-02770-s001.zip › foods-1878259-supplementary.pdf]

Supplementary Materials

The Impact of Storage Conditions and Bottle Orientation on the Evolution of Phenolic and Volatile Compounds of Vintage Port Wine

Joana Azevedo [1,*,†], Joana Pinto [2,3,*,†], Natércia Teixeira [1], Joana Oliveira [1], Miguel Cabral [4], Paula Guedes de Pinho [2,3], Paulo Lopes [4], Nuno Mateus [1] and Victor de Freitas [1]

[1] LAQV–REQUIMTE/Laboratório Associado para a Química Verde, Faculdade de Ciências, Universidade do Porto, Rua do Campo Alegre, 687, 4169-007 Porto, Portugal
[2] Associate Laboratory i4HB—Institute for Health and Bioeconomy, Faculdade de Farmácia, Universidade do Porto, Rua Jorge Viterbo Ferreira, 228, 4050-313 Porto, Portugal
[3] UCIBIO–REQUIMTE, Laboratório de Toxicologia, Departamento de Ciências Biológicas, Faculdade de Farmácia, Universidade do Porto, Rua Jorge Viterbo Ferreira, 228, 4050-313 Porto, Portugal
[4] Amorim Cork S.A., Rua dos Corticeiros 830, 4536-904 Santa Maria de Lamas, Portugal
* Correspondence: joana.azevedo@fc.up.pt (J.A.); jipinto@ff.up.pt (J.P.); Tel.: +351-220-402-700 (J.A.); +351-220-428-599 (J.P.)
† These authors contributed equally to this work.

Table S1. Color and phenolic parameters of Vintage Port wine stored under different cellar conditions and bottle orientation at 6-, 15- and 44-months post-bottling (n=5 per group at each time point).

Compound	T= 0	6 months			15 months			44 months		
		AV	AH	BH	AV	AH	BH	AV	AH	BH
L*	20.2	12.6 (6.7)	12.8 (3.6)	13.4 (3.7)	19.3 (5.9)	20.1 (3.3)	17.6 (0.9)	30.0 (1.7)	30.6 (2.3)	28.1 (5.9)
a*	48.7	36.4 (10.0)	38.2 (4.7)	37.9 (3.9)	47.6 (7.1)	49.3 (3.7)	46.3 (1.3)	48.8 (1.5)	49.8 (2.1)	47.0 (6.6)
b*	28	16.0 (6.6)	19.7 (3.1)	21.1 (3.7)	26.4 (2.6)	26.6 (0.9)	26.4 (1.3)	19.1 (0.7)	18.2 (2.1)	25.1 (13.7)
C*	56	40.9 (12.9)	43.9 (6.6)	43.6 (6.8)	54.9 (8.2)	55.9 (3.5)	52.9 (1.4)	52.3 (7.9)	53.1 (1.8)	50.3 (5.5)
Hº	29.6	25.8 (4.7)	27.5 (3.0)	27.9 (2.8)	29.0 (1.7)	28.1 (1.2)	29.7 (0.8)	21.2 (7.8)	20.1 (2.4)	20.6 (1.5)
Chromatic Intensity (CI)	29.7	38.8 (9.5)	38.0 (3.8)	41.3 (4.7)	38.8 (9.5)	38.0 (3.8)	25.9 (1.5)	26.1 (0.5)	28.3 (2.5)	26.6 (1.0)
Hue (Abs 420 nm/ Abs 520 nm)	0.58	0.74 (0.08)	0.79 (0.05)	0.70 (0.04)	0.64 (0.06)	0.61 (0.02)	0.63 (0.03)	0.72 (0.06)	0.65 (0.14)	0.78 (0.06)
Total Phenolic Index (280 nm)	102	91.3 (6.6)	82.7 (5.4)	84.9 (11.4)	77.3 (15.0)	94.1 (23.9)	84.0 (10.6)	68.4 (2.5)	71.2 (2.7)	72.2 (1.1)
Total Proanthocyanidins (g/L)	3.2	2.8 (0.3)	3.1 (0.3)	3.3 (0.2)	2.9 (0.3)	3.5 (0.3)	3.3 (0.2)	1.3 (0.5)	1.3 (0.1)	0.8 (0.5)
Anthocyanins (mg/L)	354	367.8 (36.4)	312.4 (27.2)	344.9 (59.0)	213.8 (20.3)	211.3 (17.4)	186.9 (22.4)	102.2 (29.4)	128.5 (14.7)	113.6 (8.5)
Dialysis Index (g/L)	4.9	5.6 (0.3)	4.9 (0.3)	5.2 (0.9)	4.9 (1.0)	5.4 (1.1)	5.2 (0.8)	5.5 (0.5)	5.2 (0.5)	5.5 (0.3)
Tannin Specific Activity (NTU/mL)	404	392.1 (10.6)	360.8 (13.0)	375.1 (12.5)	361.1 (9.4)	342.1 (12.0)	347.8 (19.3)	411.1 (17.2)	471.5 (29.2)	436.3 (15.3)

AV – cellar A vertical position, AH – cellar A horizontal position, BH – cellar B horizontal position

Table S2. List of the 36 volatile compounds quantified in Vintage Porto wine under different cellar conditions and bottle orientation at 6-, 15- and 44-months post-bottling (n=5 per group at each time point).

Compound	RT	*m/z*	T= 0	6 months			15 months			44 months		
				AV	AH	BH	AV	AH	BH	AV	AH	BH
Alcohols												
Isoamyl alcohol (mg/L)	3.79	55	356	446 (114)	511 (148)	426 (147)	92 (4)	96 (17)	89 (5)	447 (45)	450 (11)	416 (22)
3-Hexen-1-ol (µg/L)	6.24	67	58.6	74.5 (92.7)	88.6 (75.9)	90.3 (78.3)	51.3 (10.3)	50.9 (10.1)	50.7 (9.4)	90.7 (9.7)	95.5 (9.3)	90.6 (10.1)
1-Hexanol (mg/L)	6.60	56	0.62	0.64 (0.79)	0.92 (0.85)	0.93 (0.62)	1.14 (0.11)	1.10 (0.16)	1.08 (0.10)	26.4 (1.9)	26.7 (1.6)	24.7 (0.8)
1-Octanol (µg/L)	12.51	56	18.7	10.3 (12.8)	13.4 (11.9)	16.0 (10.8)	16.0 (1.6)	15.8 (2.5)	15.4 (1.3)	57.7 (2.4)	59.4 (2.8)	58.7 (2.7)
Phenylethyl alcohol (mg/L)	13.77	91	20.5	16.3 (15.0)	17.5 (13.5)	21.2 (11.0)	12.9 (1.6)	13.4 (1.5)	13.0 (2.0)	42.5 (9.7)	49.8 (13.1)	53.8 (3.8)
1-Decanol (µg/L)	18.33	55	1.60	18.9 (6.3)	BLOQ	15.0 (12.6)	3.0 (0.3)	3.3 (0.5)	2.7 (0.2)	67.6 (0.3)	67.2 (0.9)	68.1 (0.6)
Aldehydes												
Furfural (µg/L)	5.66	95	137	207 (74)	227 (98)	308 (118)	298 (26)	359 (92)	461 (43)	521 (90)	471 (93)	914 (43)
5-Methylfurfural (µg/L)	9.24	110	NQ	NQ	NQ	NQ	NQ	NQ	NQ	43.5 (2.0	42.8 (2.8)	64.5 (3.9)
Benzaldehyde (µg/L)	9.19	77	45.4	34.4 (10.4)	35.7 (8.3)	33.9 (8.8)	16.3 (1.9)	17.4 (1.0)	15.0 (0.2)	32.7 (4.7)	25.4 (2.9)	28.9 (3.0)
Phenylacetaldehyde (µg/L)	11.66	91	64.1	29.7 (8.3)	32.5 (7.5)	28.4 (4.5)	18.6 (3.4)	20.0 (3.0)	18.3 (1.4)	101 (11.1)	98.9 (7.8)	110 (7.0)
Nonanal (µg/L)	13.48	57	0.96	11.3 (4.5)	9.6 (3.0)	7.8 (4.0)	2.7 (1.9)	2.3 (0.6)	2.6 (0.9)	69.1 (5.1)	51.5 (11.6)	72.6 (39.9)
Decanal (µg/L)	16.46	57	3.12	14.5 (23.8)	3.6 (3.0)	5.9 (6.3)	4.0 (2.9)	3.3 (0.4)	3.0 (0.5)	14.9 (4.0)	15.1 (0.9)	23.9 (13.4)
Ethyl esters												
Ethyl isobutyrate (µg/L)	4.06	71	68.9	139 (39)	151 (56)	142 (52)	BLOQ	BLOQ	BLOQ	292 (49)	294 (58)	271 (42)
Ethyl butanoate (µg/L)	4.90	71	258	156 (44)	173 (66)	146 (54)	67 (19)	93 (27)	63 (26)	240 (34)	245 (43)	216 (30)
Ethyl 2-methylbutanoate (µg/L)	6.05	57	9.2	14.5 (4.2)	15.4 (5.3)	15.0 (5.0)	9.9 (2.2)	12.5 (4.0)	9.4 (3.2)	46.0 (6.2)	46.6 (8.3)	51.8 (9.0)
Ethyl isovalerate (µg/L)	6.16	88	9.7	24.7 (7.2)	27.3 (11.0)	25.2 (9.0)	17.8 (3.3)	22.1 (6.0)	18.2 (7.3)	109 (13.4)	111 (24.4)	112 (20.3)
Isoamyl acetate (µg/L)	6.75	70	611	284 (72)	319 (121)	238 (78)	96 (18)	114 (32)	74 (22)	148 (15)	153 (25)	129 (20)
Hexyl acetate (µg/L)	10.69	56	33.8	6.34 (1.31)	7.36 (2.79)	4.96 (1.58)	2.52 (0.42)	3.04 (0.80)	1.92 (0.44)	3.44 (0.31)	3.31 (0.80)	BLOQ
Ethyl hexanoate (mg/L)	10.26	88	0.96	0.42 (0.11)	0.49 (0.19)	0.38 (0.14)	0.19 (0.03)	0.22 (0.06)	0.16 (0.04)	1.16 (0.09)	1.20 (0.22)	1.11 (0.18)
Ethyl heptanoate (µg/L)	13.25	88	2.70	5.23 (1.81)	5.96 (2.63)	4.50 (2.09)	2.41 (0.33)	2.85 (0.64)	2.28 (0.53)	5.77 (0.53)	6.59 (1.40)	5.94 (0.99)
Diethyl succinate (mg/L)	15.71	101	NQ	NQ	NQ	NQ	3.2 (04)	3.4 (0.1)	3.9 (0.4)	43.6 (2.0)	44.4 (7.1)	54.5 (3.0)
Ethyl octanoate (mg/L)	16.27	88	NQ	NQ	NQ	NQ	0.60 (0.09)	0.74 (0.28)	0.52 (0.10)	3.66 (0.58)	3.96 (0.87)	3.46 (0.84)
Phenylethyl acetate (µg/L)	17.85	104	NQ	NQ	NQ	NQ	14.6 (1.3)	16.0 (0.8)	13.2 (1.3)	32.2 (1.1)	32.8 (1.7)	30.8 (1.2)
Ethyl nonanoate (µg/L)	18.93	88	1.8	3.0 (1.8)	3.3 (1.8)	2.8 (2.0)	3.6 (0.4)	4.2 (1.3)	3.2 (0.2)	17.7 (2.4)	19.2 (2.5)	18.2 (3.0)
Ethyl decanoate (µg/L)	21.55	88	2042	828 (377)	909 (425)	791 (486)	382 (72)	559 (374)	295 (23)	300 (115)	364 (128)	296 (119)
Ketones												

Compound	RT	*m/z*	T= 0	6 months			15 months			44 months		
				**AV**	**AH**	**BH**	**AV**	**AH**	**BH**	**AV**	**AH**	**BH**
2-Heptanone (µg/L)	7.10	58	0.66	1.95 (0.20)	2.11 (0.47)	1.87 (0.29)	0.93 (0.16)	1.06 (0.17)	0.93 (0.13)	1.01 (0.09)	1.16 (0.25)	1.03 (0.19)
2-Nonanone (µg/L)	13.04	58	1.82	1.03 (0.61)	1.21 (0.43)	1.05 (0.34)	0.67 (0.04)	0.77 (0.18)	0.63 (0.04)	2.31 (0.20)	2.42 (0.27)	2.27 (0.17)
2-Undecanone (µg/L)	18.85	58	0.972	2.96 (4.32)	1.27 (1.54)	5.28 (5.87)	0.21 (0.05)	0.23 (0.04)	0.18 (0.04)	0.98 (0.31)	1.07 (0.20)	0.74 (0.23)
Isoprenoids												
α-Pinene (µg/L)	8.36	93	2.43	1.91 (1.84)	1.21 (0.93)	0.82 (1.55)	BLOQ	BLOQ	BLOQ	ND	ND	ND
Limonene (ng/L)	11.20	68	3.39	7.25 (3.20)	9.90 (4.41)	5.47 (1.14)	NQ	NQ	NQ	31551 (1777)	31363 (2281)	30638 (3031)
Eucalyptol (ng/L)	11.28	81	ND	BLOQ	BLOQ	436 (232)	148 (17)	140 (15)	128 (23)	BLOQ	BLOQ	BLOQ
Linalool oxide (µg/L)	12.50	59	15.6	BLOQ	BLOQ	17.1 (14.0)	30.6 (5.5)	33.8 (7.6)	32.1 (3.4)	54.4 (20.9)	59.0 (14.2)	82.8 (15.5)
Linalool (µg/L)	13.33	71	30.3	24.6 (2.6)	24.1 (7.1)	18.4 (6.0)	11.2 (0.7)	13.0 (3.9)	9.7 (0.5)	23.6 (2.9)	104 (95)	30.0 (19.7)
β-Cyclocitral (ng/L)	16.84	67	56.6	72.2 (66.6)	147 (124)	11.0 (13.6)	294 (13)	332 (39)	345 (22)	BLOQ	BLOQ	BLOQ
β-Damascenone (µg/L)	21.15	69	3.63	3.02 (1.57)	2.98 (1.30)	2.87 (1.20)	1.10 (0.09)	1.36 (0.25)	1.10 (0.10)	0.013 (0.0002)	0.013 (0.0003)	0.013 (0.002)
α-Ionone (ng/L)	22.19	121	81.3	BLOQ	BLOQ	BLOQ	41.6 (2.3)	44.3 (4.9)	45.6 (2.3)	BLOQ	BLOQ	BLOQ

RT – retention time, *m/z* – mass to charge ratio, T= 0 – sample analysed at the time of bottling, AV – cellar A vertical position, AH – cellar A horizontal position, BH – cellar B horizontal position, BLOQ – below limit of quantification, ND – not detected, NQ – not quantified.

Table S3: List of Vintage Port wine bottles where Corklin was detected at 44 months post-bottling.

Wine	Corklin- *m/z* 1193 (Signal obtained with LC/MS analysis)
**Cellar A - Horizontal**	
Bottle 1	+
Bottle 2	-
Bottle 3	+
Bottle 4	+
Bottle 5	++
**Cellar B - Horizontal**	
Bottle 1	++
Bottle 2	+
Bottle 3	+
Bottle 4	-
Bottle 5	+
**Cellar A - Vertical**	
Bottle 1	-
Bottle 2	-
Bottle 3	-
Bottle 4	-
Bottle 5	-

"+" mean trace amounts detected, "- "mean non-detected.
